# Towards visible-wavelength passively mode-locked lasers in all-fibre format

**DOI:** 10.1038/s41377-020-0305-0

**Published:** 2020-04-14

**Authors:** Jinhai Zou, Chuchu Dong, Hongjian Wang, Tuanjie Du, Zhengqian Luo

**Affiliations:** Department of Electronic Engineering, Xiamen University, 361005 Xiamen, China

**Keywords:** Fibre lasers, Ultrafast lasers, Mode-locked lasers, Nonlinear optics

## Abstract

Mode-locked fibre lasers (MLFLs) are fundamental building blocks of many photonic systems used in industrial, scientific and biomedical applications. To date, 1–2 μm MLFLs have been well developed; however, passively mode-locked fibre lasers in the visible region (380–760 nm) have never been reported. Here, we address this challenge by demonstrating an all-fibre visible-wavelength passively mode-locked picosecond laser at 635 nm. The 635 nm mode-locked laser with an all-fibre figure-eight cavity uses a Pr/Yb codoped ZBLAN fibre as the visible gain medium and a nonlinear amplifying loop mirror as the mode-locking element. First, we theoretically predict and analyse the formation and evolution of 635 nm mode-locked pulses in the dissipative soliton resonance (DSR) regime by solving the Ginzburg-Landau equation. Then, we experimentally demonstrate the stable generation of 635 nm DSR mode-locked pulses with a pulse duration as short as ~96 ps, a radio-frequency signal-to-noise ratio of 67 dB and a narrow spectral bandwidth of <0.1 nm. The experimental results are in excellent agreement with our numerical simulations. In addition, we also observe 635 nm noise-like pulse operation with a wide (>1 nm) and modulated optical spectrum. This work represents an important step towards miniaturized ultrafast fibre lasers in the visible spectral region.

## Photonics: Lighting the way ultrafast compact visible light lasers

Chinese scientists have demonstrated a low-cost and compact ultrafast passively mode-locked laser that operates in the visible light spectrum and could see use in a range of industrial, scientific, and biomedical applications. Although Mode-locked fibre lasers (MLFLs) are the fundamental building blocks of many photonic systems, ultrafast lasers in the visible light spectral region are costly and challenging to make. For the first time, Zhengqian Luo and colleagues from Xiamen University in China have demonstrated a visible-wavelength passively mode-locked all-fibre laser that operates in the dissipative soliton resonance regime. The laser generates picosecond pulses of light at 635 nanometres and represents an essential step towards miniaturized ultrafast fibre lasers in the visible light range. The work lays the foundations for photonic devices for use in applications such as material processing, medicine, spectroscopy, and optical communications.

## Introduction

Mode-locked fibre lasers generating ultrashort pulses with the advantages of robustness, compactness and excellent beam quality are of tremendous interest in applications, such as material processing, medicine, spectroscopy, optical communication and scientific research^1–4^. In recent decades, mode-locked ultrafast fibre lasers operating in the near-infrared and mid-infrared spectral regions have been well developed^5–14^, but ultrafast laser sources in the visible spectral region (380–760 nm) still heavily rely on Ti:sapphire mode-locked oscillator and optical parametric amplification systems^15^ (or frequency doubling of near-infrared ultrafast lasers^16^), suffering from a large footprint and an extremely high cost. Researchers desire an alternative ultrafast visible laser solution that is compact, low cost, user friendly and maintenance free. Passive mode locking in all-fibre format could satisfy all these demands, and therefore, there is strong research motivation to develop passively mode-locked fibre lasers in the visible region.

Little research progress in visible-wavelength passively mode-locked fibre lasers has been made over the last two decades. The main challenges are as follows: (1) The fabrication of low-loss visible gain fibres is relatively arduous, and almost all visible gain fibres with fluoride-glass host material cannot be low-loss fusion spliced with other fibres, obstructing the visible all-fibre format. (2) Fibre-compatible visible mode-lockers (e.g. visible-available saturable absorbers) are lacking. (3) Visible-wavelength fibre components (fibre isolators, WDMs, couplers, high-power pump sources, etc.) are relatively immature, also restricting the realization of all-fibre visible lasers to a certain extent. (4) The ultralarge normal dispersion of the fibre cavity at visible wavelengths greatly increases the difficulty of passive mode locking. Due to the rapid development of low-loss soft-glass fibres^17^ (e.g. ZBLAN fibre) and high-power blue laser diodes (LDs) in recent years, rare-earth-doped (e.g. Pr^3+^ and Ho^3+^) ZBLAN fibres can provide high-performance optical gain at visible wavelengths^18–20^. Meanwhile, rapid progress has also been made in visible-wavelength passive fibre components and visible-available nanomaterial-based saturable absorbers (SAs, e.g. graphene^21,22^, TMDs^23,24^ and CNTs^25^). In particular, a new operation regime, dissipative soliton resonance (DSR), was proposed to overcome the difficulty of passive mode locking in a large normal-dispersion fibre cavity^26–28^. Based on the DSR regime, ~10 μJ high-energy DSR pulses in a normal-dispersion Er/Yb codoped fibre laser at 1.55 μm have been successfully achieved^29^, and a strong normal-dispersion Nd-doped fibre laser at the 927 nm short wavelength has also been mode locked^30^. Furthermore, if one can well combine the DSR regime with the abovementioned progress in the visible region, then a breakthrough in visible-wavelength passively mode-locked fibre lasers can be expected.

In this paper, we propose and demonstrate a visible-wavelength passively mode-locked all-fibre laser. First, we numerically predict the pulse formation and evolution of a 635 nm passively mode-locked Pr^3+^/Yb^3+^ codoped fibre laser operating in the DSR regime by solving the Ginzburg-Landau equation. Subsequently, we experimentally demonstrate the generation of 635 nm DSR pulses in the Pr^3+^/Yb^3+^ codoped all-fibre laser passively mode-locked by a nonlinear amplifying loop mirror (NALM). The rectangular DSR pulses with a central wavelength of ~635 nm have an ultranarrow optical spectral bandwidth (<0.1 nm) and a tuneable pulse duration of 96–1298 ps. The stable mode-locking operation has an excellent signal-to-noise ratio (SNR) of 67 dB and a repetition rate of 3.8713 MHz. The experimental results are in excellent agreement with our numerical simulations, further confirming that the 635 nm mode-locked laser operated in the resonance regime. In addition, by adjusting the intracavity polarization state, 635 nm noise-like pulses with a 590–1434 ps pulse duration and a wide (>1 nm) optical spectrum are also observed. This work represents a new paradigm in the generation of visible-wavelength ultrafast lasers for diverse potential applications in laser material processing, visible optical communication, and direct ultrafast ultraviolet generation by frequency doubling.

## Results

### Numerical simulations

The numerical model of the 635 nm passively mode-locked fibre laser is in accord with our experimental configuration. A Pr^3+^/Yb^3+^ codoped ZBLAN fibre provides ~635 nm visible-light gain, and the mode-locking element is an NALM that induces a periodic saturable absorption effect. The parameters of each component in the laser cavity are listed in Supplementary Table S1, including the nonlinear coefficient (*γ*), group velocity dispersion (GVD) coefficient (*β*_2_), gain bandwidth (Ω_g_), saturation power (*P*_sat_) and small-signal gain coefficient (*g*_*0*_). The 635 nm pulse propagation in the fibre laser can be governed by the scalar complex cubic-quintic Ginzburg-Landau equation (CGLE) and numerically solved by the standard split-step Fourier method (see Materials and methods for more details). The simulation starts with a 1-ps Gaussian pulse as the initial pulse, and one expects that the pulse may quickly converge to a steady solution in stable mode locking.

**Fig. 1:**
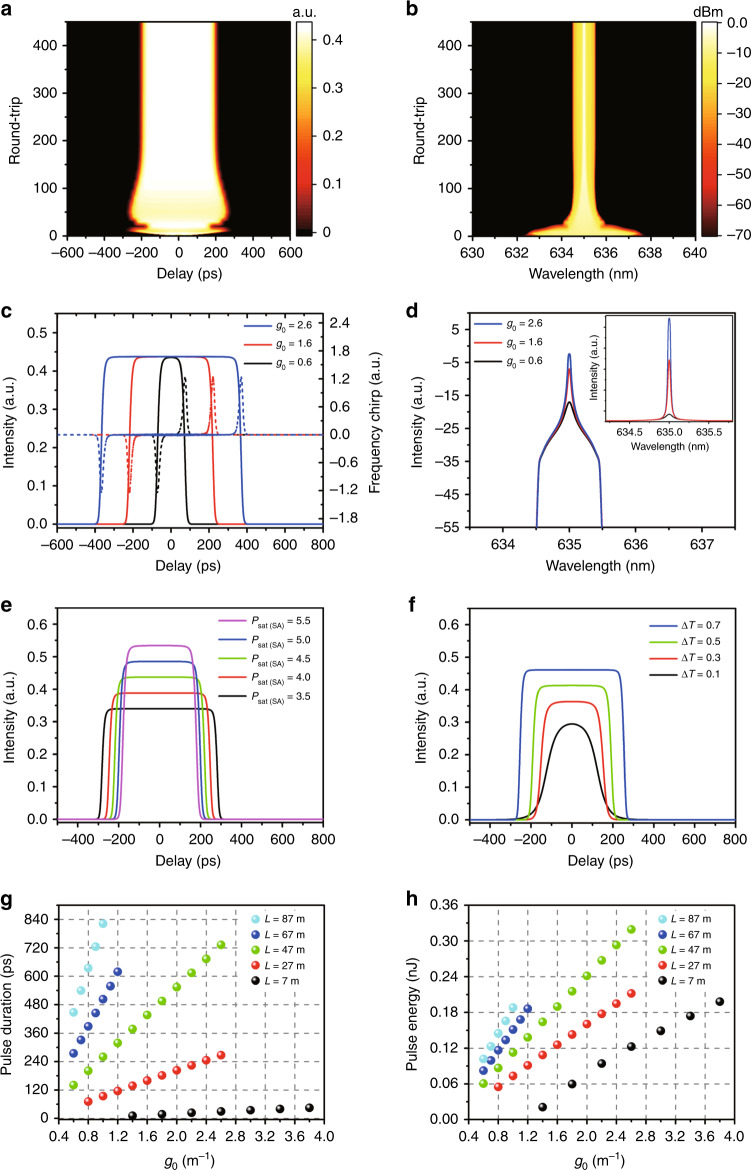
Numerical simulation results with *g*_*0*_ = 1.6 m^−1^, Δ*T* = 0.6, *P*_sat(SA)_ = 5.5 W and a fibre loop length of 47 m. **a** Pulse evolution and **b** optical spectral evolution. **c** Pulse temporal profiles (solid) and frequency chirps (dashed) and **d** optical spectra (inset: spectra on a linear scale) under different *g*_*0*_. **e** Pulse temporal profiles under different *P*_sat(SA)_. **f** Pulse temporal profiles under different Δ*T*. **g** Pulse duration and **h** pulse energy under different *L* as a function of *g*_*0*_

We simulated the formation and evolution of 635 nm mode-locked pulses in the DSR regime. The numerical results are shown in Fig. 1. With increasing number of round trips, the initial pulse quickly converges, and a stable pulse is built up in the cavity (see Fig. 1a). Because of GVD, self-phase modulation and the peak-power-clamping effect, the pulse amplitude first increases and then remains unchanged. The corresponding spectral evolution is shown in Fig. 1b. As the number of round trips increases, the optical spectrum becomes narrower, and the variation tendency of the spectral bandwidth is opposite to that of the pulse duration. Finally, the pulse, spectrum and peak power all reach their steady states even though the number of round trips further increases, indicating that 635 nm stable mode locking can be established. Figure 1c plots the evolution of the 635 nm mode-locked pulse profiles (solid) and the corresponding frequency chirps (dashed) with the small-signal gain coefficient (*g*_*0*_). As *g*_*0*_ increases (equivalent to increasing the pump power), the pulse duration linearly broadens, and the pulse amplitude remains unchanged. The pulse has two different chirp features along its profile (see Fig. 1c). One is a low linear chirp across the central region, and the other is large nonlinear chirps at both edges of the pulse^31^. Figure 1d shows the corresponding optical spectra of 635 nm mode-locked pulses. The spectra exhibit an ultranarrow spectral bandwidth (<0.1 nm) around 635 nm and a triangular profile with steep edges on the logarithmic scale. As *g*_*0*_ increases, the triangular spectral edges remain unchanged while the newly generated spectral part will overlay the spectrum and form a peak. The simulation results from Fig. 1c, d show that the 635 nm mode-locked pulses have a constant amplitude, a linearly broadening pulse duration and a narrow spectral peak profile with steep edges, which are the typical features of the DSR regime^26,32^.

It is well known that NALM mode locking originates from the nonlinear interference of the counter-propagating light fields, and its saturation power (*P*_sat(SA)_) and modulation depth (Δ*T*) highly depend on the optical coupler’s splitting ratio and the fibre loop length (*L*)^33^. For example, by changing the fibre loop length from 7 m to 87 m, the saturation power will be varied from 52.8 W to 2.7 W (see Fig. S2). To optimally design our subsequent experiment on a 635 nm passively mode-locked fibre laser, it is thus necessary to simulate the effects of the NALM’s characteristics on the DSR mode-locking performance. As the saturation power (*P*_sat(SA)_) increases, the DSR pulse duration decreases (Fig. 1e), while the pulse peak power increases (see Fig. S3b) and the optical spectrum becomes wider (see Fig. S3a). As shown in Fig. 1f, the larger the modulation depth is, the wider the pulse duration that can be supported and the higher the peak power that can be obtained in the DSR regime (see Fig. S3c and S3d for more details). Moreover, as the fibre loop length *L* increases (implying larger nonlinearity accumulation and lower saturation power of the NALM), one can see from Fig. 1g that the DSR mode-locking threshold first sharply deceases and then remains unchanged (when *L* > 47 m)^34^, while the pulse duration significantly broadens. As plotted in Fig. 1h, the pulse energy and the slope conversion efficiency gradually increase with the fibre loop length *L*. It should be especially stated that the pulse peak power always remains constant once the fibre loop length *L* is fixed (see Fig. S5b) due to the peak-power-clamping effect induced by the periodic saturable absorption^33,35^. Considering the low-threshold mode locking and high-performance output, the coupler’s splitting ratio and the fibre loop length of the NALM should be designed to be 50:50 and ~47 m, respectively, according to the numerical results in Fig. 1.

### Experimental setup and results

**Fig. 2:**
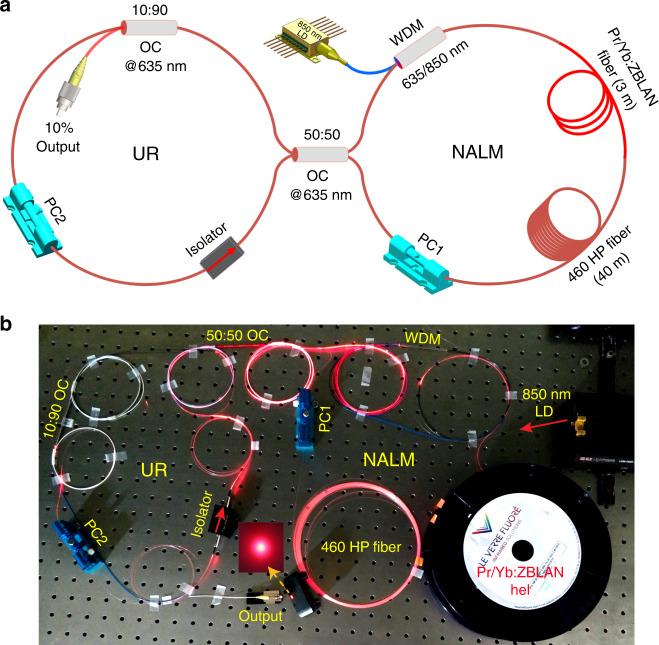
Experimental setup of the visible-wavelength passively mode-locked fibre laser. **a** Schematic and **b** photograph of the proposed 635 nm DSR mode-locked fibre laser in a figure-8 all-fibre configuration

According to the numerical simulations, we designed and further present an experimental study on a 635 nm passively mode-locked all-fibre laser. Figure 2a, b depict a schematic and a corresponding photograph of the experimental setup, respectively. The laser cavity was built in a figure-eight configuration that includes the NALM and a unidirectional ring (UR). The NALM acts as a mode-locking element and consists of a 635/850 nm wavelength division multiplexer (WDM), a 3 m Pr^3+^/Yb^3+^ codoped ZBLAN fibre, an in-line fibre polarization controller (PC1) and an ~40 m 460 HP fibre. The Pr^3+^/Yb^3+^ codoped ZBLAN fibre (ZSF SM[0.78] (Pr3000, Yb20000), Le Verre Fluoré, Inc.) has the following parameters: a 3000 ppm (wt.) Pr^3+^ doping concentration, a 20,000 ppm (wt.) Yb^3+^ concentration, an absorption coefficient of ~4.0 dB/m at 850 nm, a 0.23 numerical aperture and 2.8/125 μm core/cladding diameters. An 850 nm laser diode (LD) with a single-mode fibre pigtail is used to pump the ZBLAN fibre by the 635/850 nm WDM, providing ~635 nm strong upconversion gain through the cooperative energy transfer (ET) between the Pr^3+^ and Yb^3+^ ions^36^. The excitation pathway is as follows: (1) the ^2^F_5/2_ level of Yb^3+^ is excited by ground state absorption with 850 nm pumping, (2) ET occurs from the ^2^F_5/2_ level of Yb^3+^ to the ^1^G_4_ level of Pr^3+^, and 3) Pr^3+^ ions are then excited to the ^3^P_0_ level, which can generate strong emission at approximately 635 nm by ^3^P_0_ → ^3^H_6_. The UR comprises a 10:90 output optical coupler (OC) at approximately 635 nm, an in-line fibre PC2 and a 635 nm fibre-based optical isolator. A 50:50 OC connects the NALM to the passive UR. The 50:50 OC and ~40 m long 460 fibre with a high nonlinearity and a large normal dispersion at 635 nm were chosen to ensure a large modulation depth and a low saturation power of the NALM, which helps the formation of DSR mode locking. The cavity round-trip length is ~53 m, and the net cavity dispersion is estimated to be ~3.1 ps^2^ in the strong normal-dispersion region. In addition, we made a patch cord of the Pr/Yb codoped ZBLAN fibre and highly efficiently connected it to single-mode silicate fibre (e.g. 460 HP fibre) by a fibre adapter, guaranteeing a compact visible all-fibre cavity. The output optical spectrum was measured by a 350–1750 nm optical spectrum analyser (Ando AQ-6315E). The temporal characteristics of the visible mode-locked laser were recorded by a 12.5 GHz photodetector (ET-4000F, Electro-Optics Technology, Inc.) together with a 40 GSa/s high-speed digital storage oscilloscope with a 12-GHz bandwidth (Agilent Infiniium DSO81204A) or an electrical spectrum analyser.

**Fig. 3:**
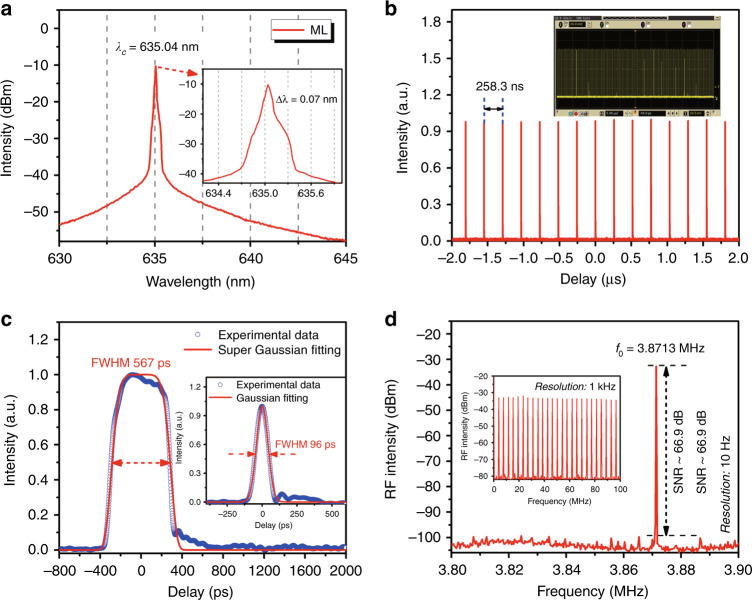
Characteristics of the 635 nm mode-locked laser under a pump power of 93 mW. **a** Output optical spectra of mode-locked (ML) operations. **b** Typical oscilloscope trace of pulse trains. **c** Single-pulse envelope (inset: single-pulse envelope under a pump power of 68 mW). **d** RF spectrum at the fundamental frequency (inset: broadband RF spectrum (100 MHz span))

In our experiment, the continuous-wave (CW) red laser threshold is 58 mW. When the pump power exceeds 68 mW, stable mode locking can be observed. The output characteristics of the red-light mode locking under the pump power of 93 mW are illustrated in Fig. 3. As shown in Fig. 3a, the mode-locked optical spectrum has a central wavelength of 635.04 nm with a 3-dB bandwidth of ~0.07 nm and exhibits a narrow spectral peak on a triangular pedestal profile (similar to the numerical simulation in Fig. 1d). Figure 3b plots the typical pulse trains with an interval of ~258.3 ns, which well matches the cavity round-trip time (corresponding to the cavity length of ~53 m). The output pulses have uniform intensity (see the inset of Fig. 3b), indicating that the mode-locked red laser is very stable (also see Supplementary Video S1). Figure 3c shows the single-pulse envelope, and the pulse has a flat-top rectangular profile and a 567 ps pulse duration if a super-Gaussian fitting is used. It is worth noting that the minimum pulse duration of 96 ps was observed at the pump power of 68 mW (see the inset of Fig. 3c). In addition, as depicted in Fig. 3d, the output radio-frequency (RF) spectrum, which was recorded with a 10 Hz resolution bandwidth, has a fundamental frequency of 3.8713 MHz and a high SNR of ∼66.9 dB. The inset of Fig. 3d shows no spectral modulation of the 100 MHz-span RF spectrum, further indicating stable CW mode-locking operation of the 635 nm visible fibre laser.

**Fig. 4:**
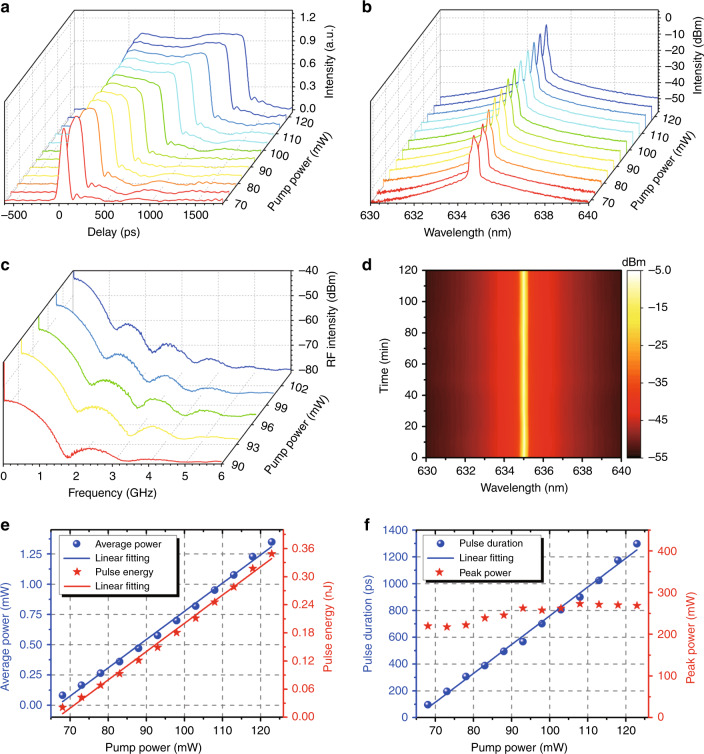
Characteristics in the 635 nm DSR regime. **a** Single-pulse envelope as the pump power increases. **b** Optical spectra versus pump power on a logarithmic scale. **c** RF spectra with a pump power from 90 to 104 mW over a wide span (6 GHz). **d** Spectral stability measurement of DSR operation under a pump power of 93 mW. **e** Average output power and pulse energy versus pump power. **f** Pulse duration and peak power as a function of the pump power

To better understand the 635 nm mode-locking operation, we further investigated the typical output features of the mode-locked fibre red laser. Figure 4a shows the single-pulse evolution with the pump power. One can see that with increasing pump power, the pulse duration gradually increases, exhibiting a typical feature of the DSR regime^27^. The corresponding optical spectrum is given in Fig. 4b, and the narrow peak on the triangular pedestal profile becomes stronger and sharper. It can be interestingly seen that the experimental results in Fig. 4a, b are in excellent agreement with the numerical results in Fig. 1c, d, further indicating that the 635 nm mode-locked laser operated in the DSR regime. Figure 4c records the broadband RF spectra over a wide span (6 GHz) under different pump powers. The periodic modulation envelope (related to the pulse duration) can be clearly observed in the RF spectra. As the pump power increases from 90 mW to 104 mW, the RF modulation period decreases from ~1.83 GHz to ~1.20 GHz, and the pulse duration correspondingly increases from ~550 ps to ~830 ps (see Fig. 4a), which agrees well with the DSR theory^37^. To evaluate the operational stability of the 635 nm DSR mode-locked all-fibre laser, we recorded the optical spectra and RF spectra every 10 min over 2 h under a pump power of 93 mW. Based on the measured data, the RMS of the RF intensity at the fundamental frequency can be evaluated to be ~0.209 dBm (corresponding to an only ~0.4% relative RF intensity fluctuation), implying stable pulse operation. Furthermore, as plotted in Fig. 4d, the central wavelength of the laser output did not drift nor did the 3-dB bandwidth vary during the 2-h test, illustrating the excellent repeatability and long-term stability. Figure 4e gives the average output power and pulse energy as a function of the pump power. Both the average power and pulse energy linearly increase without any saturation. A maximum output power of 1.35 mW was obtained, and the pulse energy was calculated to be 0.35 nJ under a pump power of 122.9 mW. The pulse duration and peak power as a function of the pump power are plotted in Fig. 4f. As the pump power increases from 68.0 mW to 122.9 mW, the pulse duration experiences a linear broadening from 96 ps to 1298 ps, whereas the peak power slightly increases initially and then remains constant. In our experiment, we tried to compress the pulse duration using a simple visible grating pair (GR25-1208, Thorlabs, Inc.), but only slight compression from 703 ps to 626 ps was obtained, possibly limited by the large nonlinear chirp of such a DSR pulse (see Fig. 1c). A higher compression ratio can be expected by specially designing a nonlinear visible grating pair^31^. Additionally, the 635 nm DSR laser with an ~567 ps duration and an average power of 0.4 mW was amplified by using a homemade Pr^3+^/Yb^3+^ codoped ZBLAN fibre amplifier (see Fig. S6a). An amplified laser output with a maximum average power of 5.1 mW, a pulse energy of 1.32 nJ and a peak power of ~2.3 W was obtained (see Fig. S6 for more details), showing the all-fibre amplification potential of such 635 nm mode-locked pulses.

**Fig. 5:**
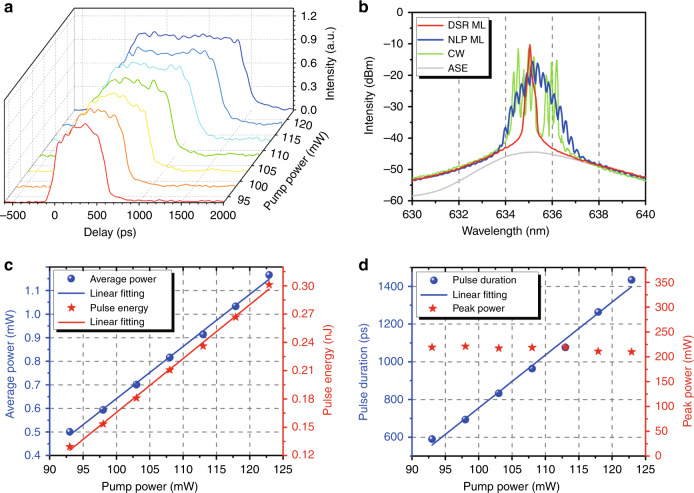
Characteristics in the 635 nm NLP regime. **a** Oscilloscope traces of the single-pulse envelope as the pump power increases. **b** Optical spectra in the CW (green), NLP mode-locking (blue) and DSR mode-locking (red) operation states. **c** Average output power and pulse energy as a function of pump power. **d** Pulse duration and peak power as a function of the pump power

A noise-like pulse (NLP), which consists of many chaotic sub-pulses with a low temporal coherence^38^, is another typical operation state in the 635 nm passively mode-locked figure-eight fibre laser. In our experiment, by changing the polarization state (i.e. rotating the PCs) and adjusting the pump power, a noise-like mode-locked pulse could also be observed. Figure 5 shows the typical characteristics in the NLP regime. As shown in Fig. 5a, although the noise-like pulse evolution with increasing pump power has similar features (i.e. a constant amplitude and pulse broadening) to the DSR pulse in Fig. 4a, the 635 nm NLP is very chaotic at the top of the rectangular pulse (see Supplementary Video S2), completely different from the stable top of the DSR pulse (see Supplementary Video S1). Figure 5b gives the output optical spectra under the different operation states of the 635 nm fibre laser. One can see that (1) under CW operation, the optical spectrum (green) is relatively wide and composed of some irregular peaks; (2) under DSR mode-locking operation, the spectrum (red) becomes very smooth and has a narrow bandwidth (<0.1 nm) spectrum with typical steep edges; and (3) under NLP mode-locking operation, the optical spectrum (blue) is obviously broader and has an increasing spectral bandwidth as the pump power increases (see Fig. S7a), which is a typical spectral characteristic of NLPs^38,39^. Moreover, a modulated optical spectrum (~0.24 nm period) emerges in the NLP mode locking, which is due to the strong nonlinear effects (e.g. self-phase modulation and four-wave mixing) in optical fibres excited by the high peak-power sub-pulses in NLPs^40^. The NLP also has a fundamental frequency of 3.8713 MHz and an SNR of ∼57.1 dB (see Fig. S7b). It should be noted that the RF spectrum in the DSR regime has a highly contrasted envelope modulation (see Fig. 4c), while the NLP is chaotic and has a low amplitude envelope modulation (see the inset in Fig. S7b). To further present the output features of the NLP, the average output power and pulse energy as a function of pump power are given in Fig. 5c. As seen from Fig. 5d, as the pump power increases from 93 mW to 122.9 mW, the pulse duration of the NLP expands from 590 ps to 1434 ps, whereas the peak power remains unchanged, which is quite similar to the case of the DSR regime.

## Discussion

In summary, we numerically and experimentally demonstrated a 635 nm all-fibre passively mode-locked laser based on an NALM for the first time. First, by solving the Ginzburg-Landau equation using the standard split-step Fourier method, the formation and evolution of 635 nm mode-locked pulses in the DSR regime were predicted. The numerical results show that as the small-signal gain coefficient increases (i.e. the pump power increases), the 635 nm rectangular pulses present a linearly broadening pulse duration, a constant amplitude and a narrow spectral peak profile with steep edges. Moreover, we also simulated the influences of the NALM’s characteristics on the DSR mode-locking performance in detail, revealing that an optimal fibre loop length (~47 m) and a large splitting ratio (50:50) of the optical coupler in the NALM should be chosen to ensure low-threshold mode locking and high-performance output. Then, based on our numerical results, we further experimentally realized a 635 nm all-fibre passively mode-locked Pr^3+^/Yb^3+^ codoped ZBLAN fibre laser. The DSR mode-locking spectrum has a central wavelength of 635.04 nm with a spectral bandwidth of <0.1 nm. The corresponding pulses have a tuneable picosecond duration (96–1298 ps) and a pulse repetition rate of 3.8713 MHz with an excellent signal-to-noise ratio (>66 dB). In addition, by adjusting the in-line fibre PCs and pump power, we also obtained NLP operation with a tuneable pulse duration from 590 ps to 1434 ps and a regularly modulated optical spectrum. The results afford a new paradigm in the generation of visible-wavelength ultrafast pulses for diverse potential applications in laser material processing and frequency doubling in the ultraviolet wavelength region.

## Materials and methods

We numerically simulated the formation and evolution of DSR in the 635 nm passively mode-locked Pr^3+^/Yb^3+^ codoped fibre laser by solving the scalar complex cubic-quintic Ginzburg-Landau equation (CGLE), which considers the most important physical effects of group velocity dispersion (GVD), self-phase modulation, and gain saturation with a finite bandwidth. The CGLE used in our simulations can be written in the following form^41^:

1 $$\frac{{\partial A}}{{\partial z}} = - \frac{{i\beta _2}}{2}\frac{{\partial ^2A}}{{\partial t^2}} + i\gamma \left| A \right|^2A + \frac{g}{2}A + \frac{g}{{2\Omega _{\mathrm{g}}^2}}\frac{{\partial ^2A}}{{\partial t^2}}$$

where *A* is the electric field envelope, *t* and *z* represent the pulse local time and propagation distance, *β*_*2*_ describes the GVD coefficient of the optical fibre, and *γ* refers to the fibre nonlinear coefficient. Ω_g_ and *g* are the gain bandwidth and gain function of the Pr^3+^/Yb^3+^ codoped fibre around 635 nm. Generally, the average power in the cavity should be considered when simulating the gain saturation of the gain fibre. The gain saturation effect is considered as follows:

2 $$g = \frac{{g_0}}{{1 + P_{{\mathrm{ave}}}/P_{{\mathrm{sat}}}}}$$

3 $$P_{{\mathrm{ave}}} = E_{\mathrm{P}}/T_{\mathrm{R}}$$

4 $$E_{\mathrm{P}} = \mathop {\int }\nolimits_{ - T_{{\mathrm{R}}/2}}^{T_{\mathrm{R}}/2} \left| {A(z,t)} \right|^2d\tau$$

where *g*_*0*_ is the small-signal gain coefficient at the central wavelength, *P*_sat_ represents the saturation power of the gain fibre, *P*_ave_ is the intracavity average power, *E*_P_ refers to the single-pulse energy, and *T*_R_ is the cavity round-trip time. According to Eq. (2), the gain of the laser cavity can be controlled by *g*_*0*_ or *P*_sat_. In our simulations, *P*_sat_ is fixed at 0.3 mW, and *g*_*0*_ is variable. In addition, the mode-locking element used in our simulation is an NALM that induces a periodic saturable absorption effect. The NALM’s power-dependent transmission coefficient curves can be well fitted by the following equation^42^:

5 $$T = \frac{1}{2}\left[ {1 - \Delta T{\mathrm{cos}}\left( {\frac{{\pi P}}{{P_{{\mathrm{sat}}\left( {{\mathrm{SA}}} \right)}}}} \right)} \right]$$

where *ΔT* is the modulation depth, *P* refers to the instantaneous pulse power and *P*_sat(SA)_ represents the saturation power of the NALM.

## Supplementary information

Supplementary Information

Supplementary Video S1

Supplementary Video S2

## Data Availability

The data that support the plots within this paper and other findings of this study are available from the corresponding author upon reasonable request.
