# Supplementary material for: Towards visible-wavelength passively mode-locked lasers in all-fibre format
Source: Light Sci Appl. 2020 Apr 14;9:61. doi: 10.1038/s41377-020-0305-0 (PMC7156699; doi:10.1038/s41377-020-0305-0)
Supplement: Supplementary file 1 — Supplementary Information [file 41377_2020_305_MOESM1_ESM.docx]

**Supplementary Information for**

Towards visible-wavelength passively mode-locked lasers in all-fibre format

Jinhai Zou^1^, Chuchu Dong^1^, Hongjian Wang^1^, Tuanjie Du^1^, and Zhengqian Luo^1, *^

^1^Department of Electronic Engineering, Xiamen University, Xiamen 361005, China

*Correspondence: Zhengqian Luo, Email: [zqluo@xmu.edu.cn](mailto:zqluo@xmu.edu.cn),

Tel: +86 15960266317, Fax: +86 592 2580041

**1. Parameters used in simulations**

**
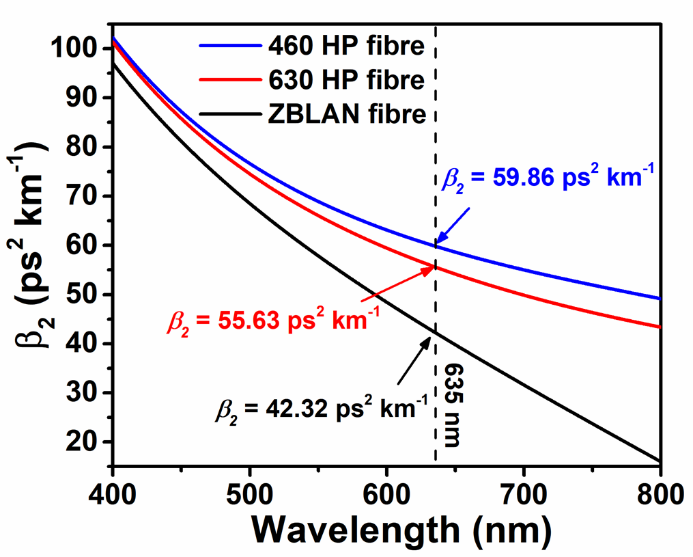
**

**Fig. S1** **Calculated** **group velocity dispersion (GVD) coefficient (*β_2_*) curves of** **the 460 HP fibre (blue), 630 HP fibre (red) and ZBLAN fibre (black).**

In our numerical simulation, the parameter values (*e.g.*, nonlinear coefficient (*γ*), group velocity dispersion coefficient (*β_2_*)) of the optical fibres (*i.e.*, 460 HP fibre, 630 HP fibre and ZBLAN fibre) is same as ones in our experimental setup of the 635 nm passively mode-locked fibre laser. According to refs.^1-3^, the *γ* values of the ZBLAN fibre, 460 HP fibre and 630 HP fibre are calculated as 66.1 W^-1^ km^-1^, 99.1 W^-1^ km^-1^ and 50.6 W^-1^ km^-1^ at 635 nm, respectively. In addition, we numerically calculated the *β_2_* values of the 460 HP fibre, 630 HP fibre and ZBLAN fibre, respectively. As plotted in **Fig. S1**, the *β_2_* values of the 460 HP fibre, 630 HP fibre and ZBLAN fibre are 59.86 ps^2^ km^-1^, 55.63 ps^2^ km^-1^ and 42.32 ps^2^ km^-1^ at our laser wavelength (635 nm), respectively.

**Table S1**

Parameters used in numerical simulations

| ZBLAN fibre | WDM | 50:50 OC | 10:90 OC | Isolator (ISO) | 460 HP fibre |
| --- | --- | --- | --- | --- | --- |
| *β*_2_ = 42.32 ps^2^ km^-1^  *γ* = 66.1 W^-1^ km^-1^  *L*_g_ = 3 m  *Ω*_g_ = 10 nm  *P*_sat_ = 0.3 mW  *g_0_*: variable | *β*_2_ = 55.63 ps^2^ km^-1^  *γ* = 50.6 W^-1^ km^-1^  *L*_WDM_ = 2 m | *β*_2_ = 55.63 ps^2^ km^-1^  *γ* = 50.6 W^-1^ km^-1^  *L*_OC1_ = 4 m | *β*_2_ = 55.63 ps^2^ km^-1^  *γ* = 50.6 W^-1^ km^-1^  *L*_OC2_ = 2 m | *β*_2_ = 55.63 ps^2^ km^-1^  *γ* = 50.6 W^-1^ km^-1^  *L*_ISO_ = 2 m  Loss = 1 dB | *β*_2_ = 59.86 ps^2^ km^-1^  *γ* = 99.1 W^-1^ km^-1^  *L*_460 HP_: variable |

The parameters of each component used in our simulations are summarized in **Table S1**. Furthermore, the insertion loss of the WDM, OC and optical fibres are negligible in the simulations because they are very small. For the nonlinear amplifying loop mirror (NALM) that induces a periodic saturable absorption effect, its saturation power (*P*_sat(SA)_) can be obtained by fitting the reflectivity *R*. The reflectivity *R* is given by:

$R=2\alpha(1-\alpha)\{1+cos[(1-2\alpha)\cdot\gamma PL]\}$ (S1)

where, *α* is the splitting ratio of the optical coupler, $\gamma$ refers to the fibre nonlinear coefficient, and *L* presents the fibre loop length of the NALM. In our simulations, an OC with a 45:55 splitting ratio (*i.e.,* *α* = 0.45) was chosen. The saturation powers of the NALM (*P*_sat(SA)_) were calculated according to Eq. (S1). As plotted in **Fig. S2**, one can been seen the *P*_sat(SA)_ decreases with the fibre loop length *L* increases, and once *L* > 47 m, the saturation power is nearly unchanged.


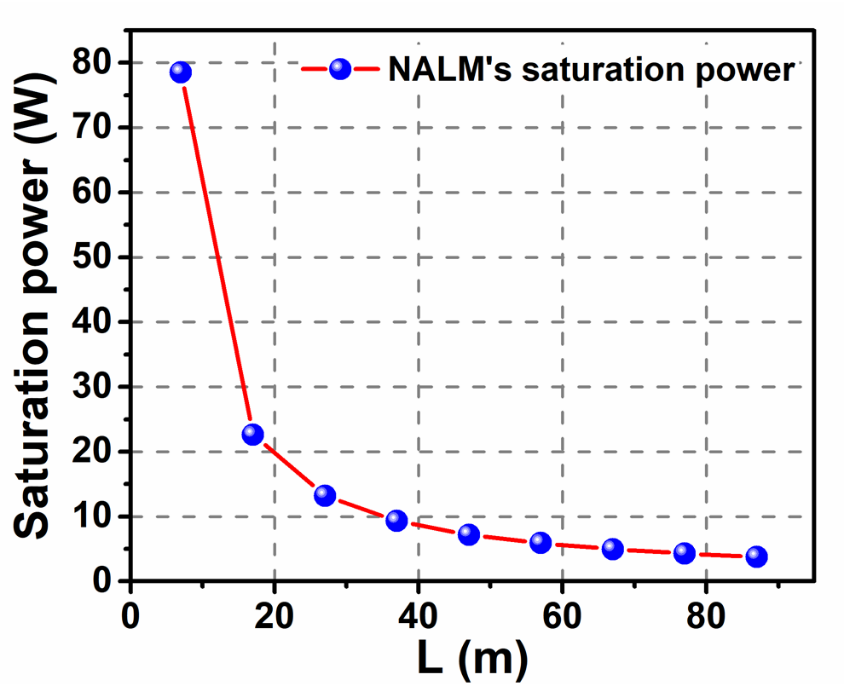


**Fig. S2 The NALM’s saturation power** **versus fibre loop length (*L*).**

**2. Simulation results**

We numerically simulate the effects of the NALM’s saturation power (*P*_sat(SA)_) and modulation depth (*ΔT*) on the DSR mode-locking performance. **Fig. S3a** and **S3b** present the optical spectrum, pulse duration and peak power versus NALM’s saturation power. As the *P*_sat(SA)_ increases, the optical spectrum becomes wider, and the pulse duration decreases, while the pulse peak power increases. As shown in **Fig. S3c** and **S3d**, with the modulation depth increases, the optical spectrum becomes narrower, the pulse duration becomes wider and the peak power is higher.


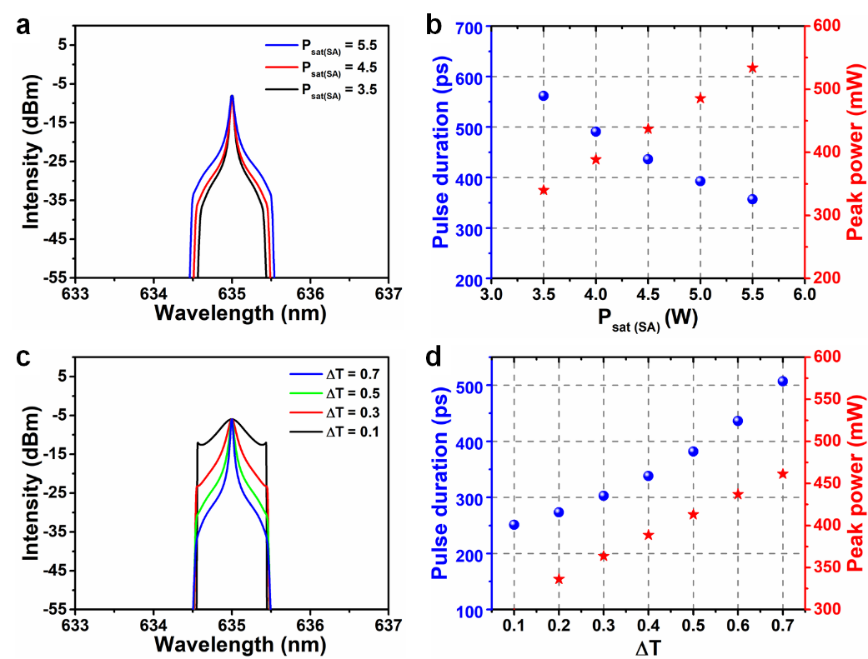


**Fig. S3 Numerical results under a fibre loop length of 47 m. a** Optical spectra, **b** pulse duration and peak power as a function of the NALM’s saturation power with *g_0_* = 1.6 m^-1^ and *ΔT* = 0.6. **c** Optical spectra, **d** pulse duration and peak power versus modulation depth (*ΔT*) with *g_0_* = 1.6 m^-1^ and *P*_sat(SA)_ = 5.5 W.

Additionally, we also investigated the effects of the NALM’s fibre loop length (*L*) on the 635 nm mode-locking output characteristics in details. As shown in **Fig. S4**, when the fibre loop length *L* increases, the mode-locking operation is easier to initiate, and the pulse duration becomes wider, while the optical spectrum becomes narrower. **Fig. S5a** shows the output power gradually increases with increasing *g_0_*, and the slope efficiency increases with the *L* increases. **Fig. S5b** plots the pulse peak powers versus *g_0_* under different *L*. We can clearly see that the peak power becomes smaller with the *L* increases, and it remains constant with increasing *g_0_* due to the peak-power-clamping effect.


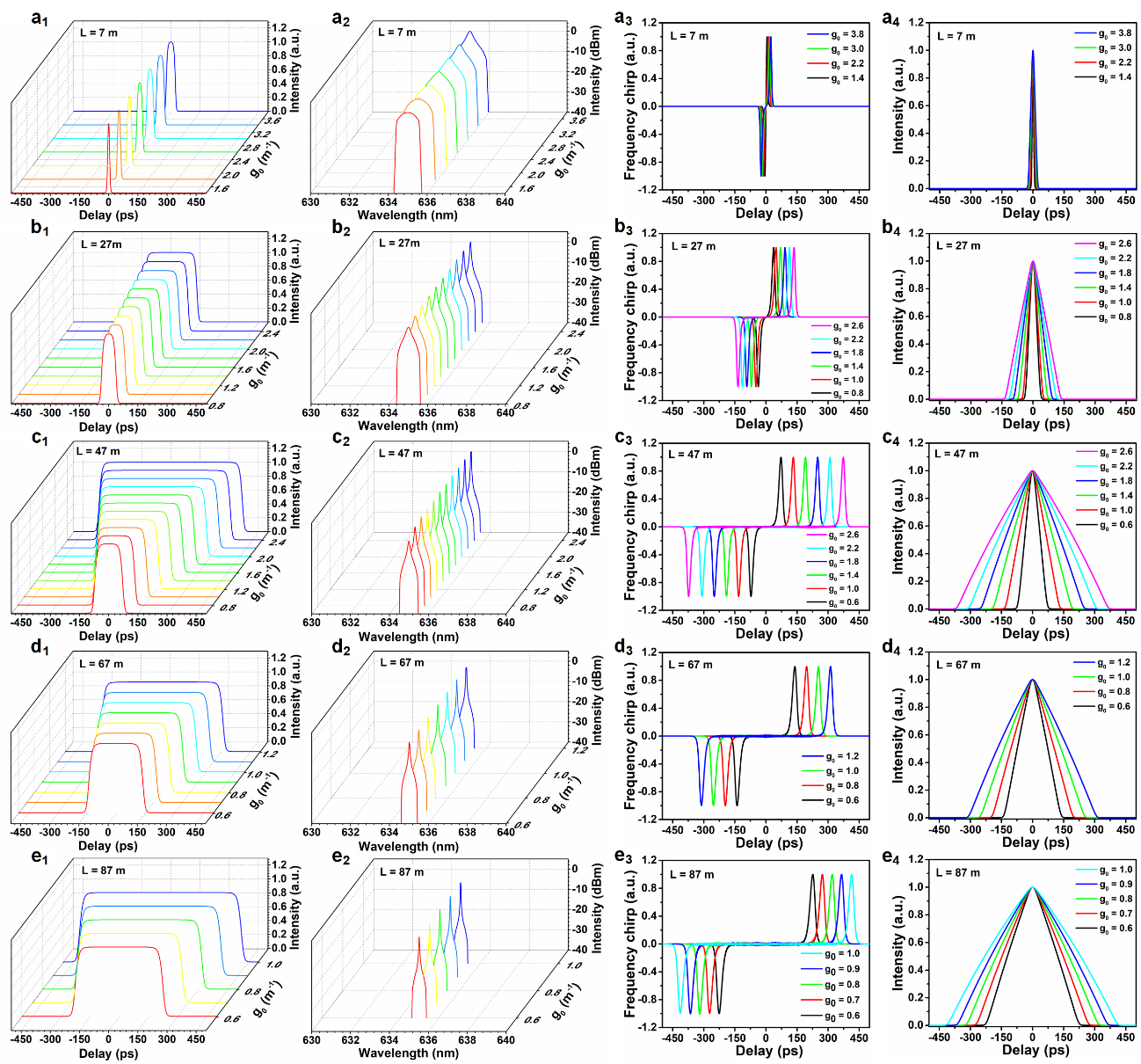
**Fig. S4 Typical numerical results versus small signal gain coefficient (*g_0_*) under different fibre loop lengths (*L*). a_1_-e_1_** Pulse temporal profiles, **a_2_-e_2_** optical spectra, **a_3_-e_3_** frequency chirps, and **a_4_–e_4_** autocorrelation traces.


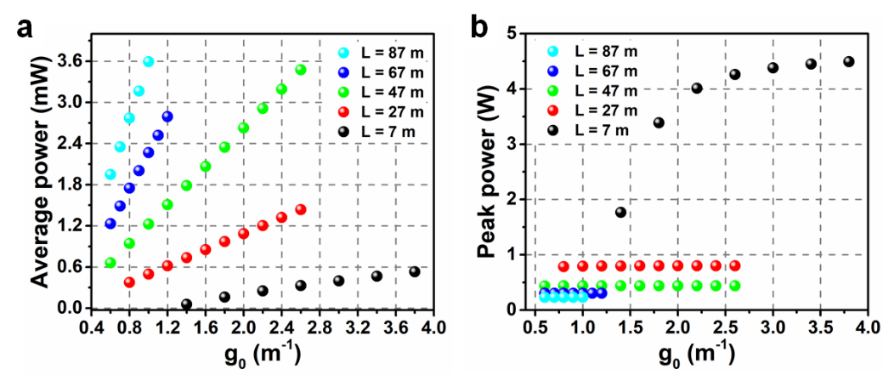


**Fig. S5 Numerical simulation results** **with increasing small signal gain coefficient (*g_0_*) under different fibre loop lengths (*L*). a** Output average power. **b** Pulse peak power.

**3. Experiment results**

**Figure S6a** depicts the schematic of the 635 nm all-fibre passively mode-locked laser amplifier. The setup simply consists of a 443/635 nm wavelength division multiplexer (WDM), a 10 cm Pr^3+^/Yb^3+^ codoped ZBLAN fibre and a 443 nm GaN laser diode (LD). The Pr^3+^/Yb^3+^ codoped ZBLAN fibre (ZSF SM [0.78] (Pr3000, Yb20000), Le Verre Fluoré, Inc.) has the following parameters: a 3000 ppm (wt.) Pr^3+^ doping concentration, a 20000 ppm (wt.) Yb^3+^ concentration, an absorption coefficient of ~4.0 dB/m at 850 nm, a 0.23 numerical aperture and 2.8/125 μm core/cladding diameters. In our experiment, an ~567 ps duration, 0.4 mW average power DSR mode-locked laser was used for amplification. The amplified output performances were plotted in **Fig. S6b-e.** As a result, with the 443 nm pump power increases from 29.8 mW to 125.1 mW, the average output power increases from 0.47 mW to 5.1 mW. Both the pulse energy and peak power linearly increase without any saturation (see **Fig. S6c**). Due to the 443 nm pump power limitation, an only 5.1 mW maximum power was obtained in our experiment and the corresponding pulse energy and peak power are calculated to be 1.32 nJ and 2.3 W. **Figure S6d** and **S6e** record the single pulse envelope and optical spectrum of the seed mode-locked laser and amplifying laser. It can be clearly seen that the pulse and spectrum before and after amplification remain almost unchanged.


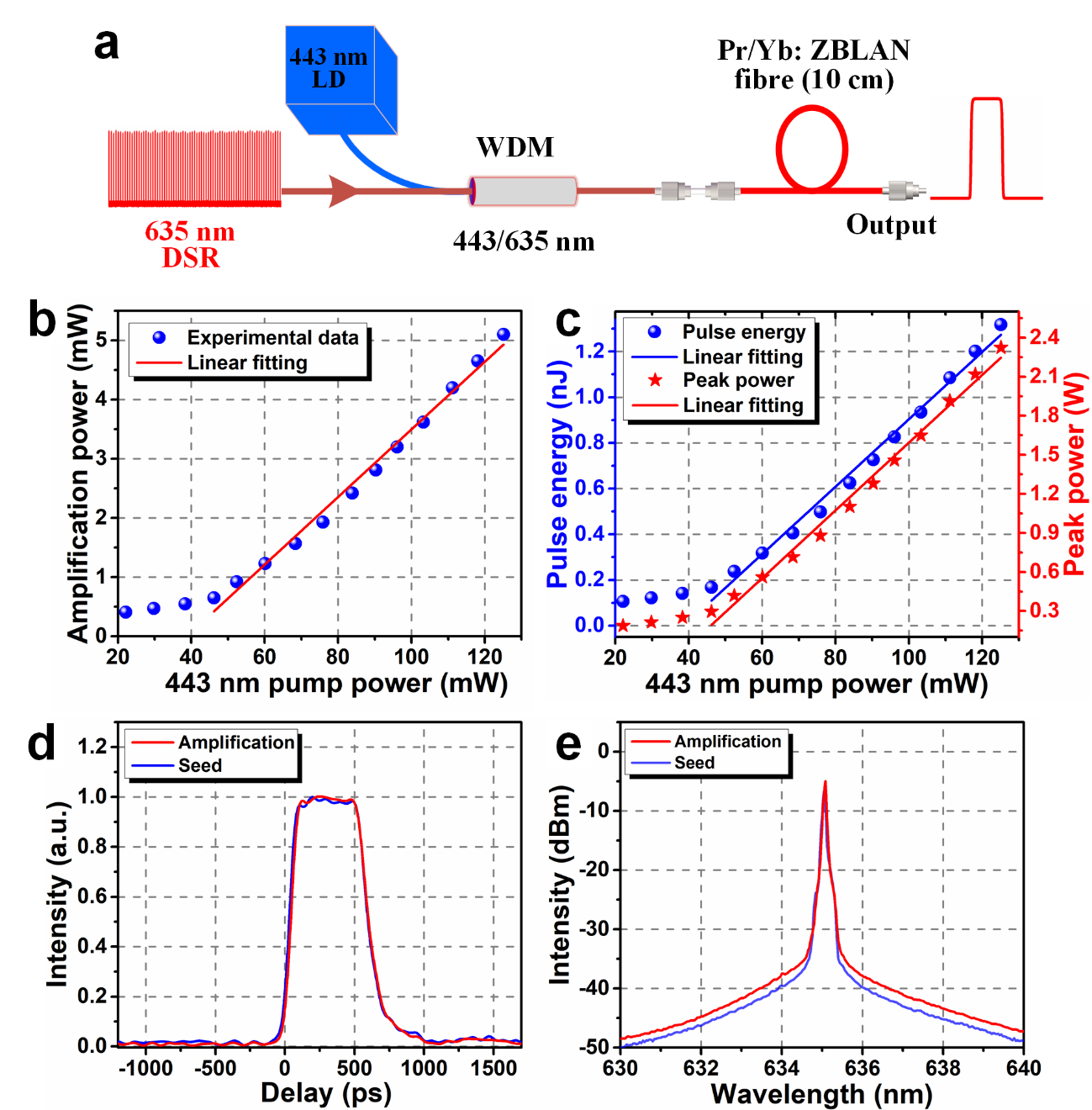


**Fig. S6 Amplification of the 635 nm DSR mode-locked laser at a seed power of 0.4 mW. a** Amplifying setup. **b** Amplifying output power versus 443 nm pump power. **c** Amplifying pulse energy and peak power with increasing 443 nm pump power. **d** Amplifying pulse and seed pulse. **e** Amplifying spectrum and seed spectrum.


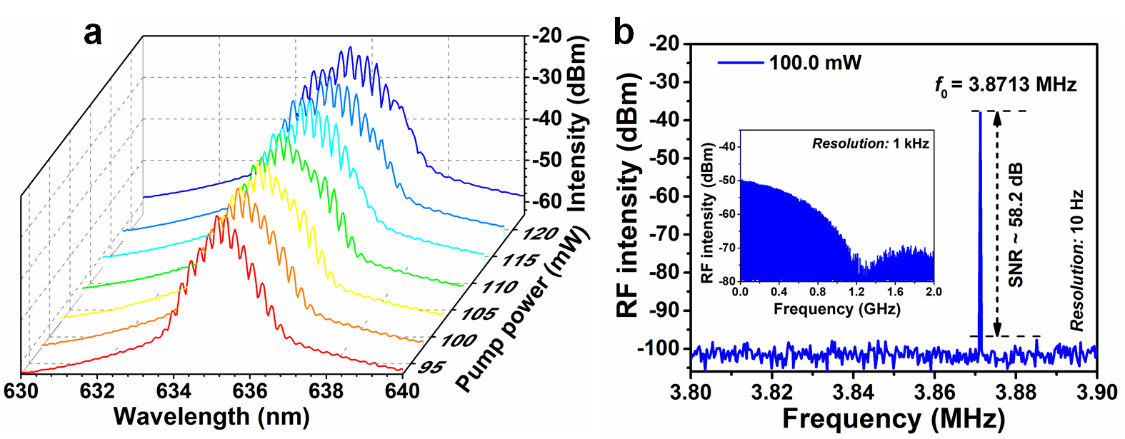


**Fig. S7 Characteristics in the 635 nm NLP regime. a** Optical spectra versus pump power on a logarithmic scale**. b** RF spectrum at the fundamental frequency under a pump power of 100 mW (inset: broadband RF spectra (2 GHz)).

**4. Supporting Videos**

**Video S1** records the dissipative soliton resonance (DSR) pulse in the 635 nm all-fibre passively mode-locked Pr^3+^/Yb^3+^ codoped ZBLAN fibre laser.

**Video S2** records the noise-like pulse (NLP) in the 635 nm all-fibre passively mode-locked Pr^3+^/Yb^3+^ codoped ZBLAN fibre laser.

**References**

1 Parker, J. M. Fluoride glasses. *Annual Review of Materials Science* **19**, 21-41 (1989).

2 Kato, T., Suetsugu, Y. & Nishimura, M. Estimation of nonlinear refractive index in various silica-based glasses for optical fibers. *Optics Letters* **20**, 2279-2281 (1995).

3 Milam, D. Review and assessment of measured values of the nonlinear refractive-index coefficient of fused silica. *Applied Optics* **37**, 546-550 (1998).
